# Observations on Bloodletting

**Published:** 1830-03

**Authors:** G. O. Heming

**Affiliations:** Member of the Royal College of Surgeons.


THE LONDON
### Medical and Physical Journal.

No. 373, VOL. LXIII.] MARCH 1830. [No. 45, *New Series.*

For many fortunate discoveries in medicine, and for the detection of numerous errors, the world is indebted to the rapid circulation of Monthly Journals; and there never existed any work, to which the Faculty, in Europe and America, were under deeper obligations than to the *Medical and Physical Journal of London*, now forming a long but an invaluable series.—*Rush.*

### ORIGINAL PAPERS, AND CASES,

### OBTAINED FROM PUBLIC INSTITUTIONS AND OTHER AUTHENTIC SOURCES.

### BLOODLETTING.

*Observations on Bloodletting.* By G. O. Heming, Esq. Member of the Royal College of Surgeons. (In a letter to Mr. North.)

Sir: Observing that you are engaged in the review of Dr. Marshall Hall's work upon the Effects of Loss of Blood, I beg to present you with the results of my experience on this subject, subsequent to the date of a paper which was inserted in the Medical Gazette, Number 93, for September 12th, 1829.

The whole of my recent trials perfectly confirm the observations of Dr. Hall, and the remarks I made in the paper to which I have alluded; and I know of no observations in the whole circle of physic of such practical importance.

I find it difficult to state whether the fact of tolerance or intolerance of loss of blood, is most useful as a guide to the diagnosis or to the treatment of disease: in both these points of view, this fact is now become indispensable to me in all cases really or apparently requiring the use of the lancet.

If I were permitted to know *but one* fact of a case, indeed, and compelled to be ignorant of all others, the fact which I would prefer to know would certainly be the degree of tolerance or intolerance of loss of blood.

Now, this fact is at once determined most accurately, by the simple plan of placing a patient erect, and bleeding from a good-sized orifice, making all due allowances for nervousness in the patient, or defective manner of taking the blood, &c.

This fact is, I venture to assert, the best, the most useful guide, to the diagnosis, and in the treatment of acute diseases. In one case of affection of the head, there appeared to be every symptom of inflammation. I bled my patient, a female twenty-four years old, and she fainted on losing eight ounces of blood. It was therefore plain that the case was *not* inflammation. I treated it by other remedies, and my patient recovered most favorably. This distinction between inflammation and irritation is one of the most important which we owe to Dr. Hall; and this mode of establishing the distinction is most simple and satisfactory. In another case, resembling peritonitis, there was a difference of opinion as to the nature of the disease. I proposed to determine it by ascertaining the degree of tolerance or intolerance of the loss of blood. The patient bore to lose a large quantity, flowing well from the vein, without syncope. The case was thenceforth considered and treated as inflammation, with the same favorable event as the former one. Indeed, nothing can be more satisfactory than this mode of proceeding.

I shall first give a list of the cases, the detail of which I propose to lay before your readers. In this manner an interesting *coup d'œil* will be presented of the remarkable phenomena of tolerance, and of intolerance, of loss of blood.

I. *Acute rheumatism;* æt. thirty, very strong; *blood lost, fifty-two and fifty-six ounces.*

II. *Pneumonia;* æt. twenty-one, not strong; *thirty-six and thirty-four ounces.*

III. *Phrenitis;* æt. ten; *eighteen ounces.*

IV. *Pneumonia;* æt. five; *seventeen ounces.*

V. *Pleuritis and tracheitis;* æt. six; *ten ounces.*

VI. *Affection of the head, not inflammation*; æt. twenty-four; *seven ounces.*

VII. *Ditto:* æt. twenty; *seven and a half, and nine ounces.*

VIII. *Hysteria;* æt. nineteen; *ten ounces.*

Case I. was that of a very strong man, and, till the present attack, his health had been always very good; he had been suffering four days with acute rheumatism when I first saw him; he was bled to fifty-two ounces, but this produced no effect on the disease, nor did it cause the slightest approach to syncope, although so large a quantity was taken. Two days after this, his pain was still very acute, and he was again bled. This time fifty-six ounces were abstracted, and he fainted. The fainting was succeeded by slight convulsions, and afterwards delirium came on, which lasted about two hours. Notwithstanding he had lost so much blood, he experienced no unpleasant symptoms from the bleeding after the first three hours: his pain left him, and did not return, and he soon regained his usual state of health.

Case II. This young man was by no means strong. He was bled forty-eight hours from the commencement of the pain, and was greatly relieved at each bloodletting. He had such symptoms as left no doubt upon my mind that it was a case of pneumonia.

Case III. was a most aggravated case of phrenitis. I had been, three weeks previous to this attack, attending this little girl with acute anasarca succeeding to scarlet fever: her symptoms were then a slight swelling of the hands, feet, face, and other parts of the body; the pulse was 120; the tongue clean, with its papillæ, particularly at the end, much raised; there was a small superficial ulcer upon each tonsil; her urine was scanty, and her bowels, unless she took opening medicine, were confined. From this state she appeared to be recovering by taking active purgatives, when she was one night suddenly seized with vomiting and violent pain in the head; but it was not till the following morning that I saw her, and I was then told that she had been found on that morning quite insensible, occasionally vomiting. She attempted to speak upon being raised, but could not articulate so that I could understand what she would have said; her face was very red and swollen; the eyes were red, but the blood-vessels of the left eye (the side on which she was lying,) were much more highly injected than those of the right; her pulse was hard, frequent, and full; her carotid arteries beat violently. She became violently convulsed whilst I stood by her, and this state was not at all altered till I made a large opening into the jugular vein, and eighteen ounces of blood had flowed rapidly from it. Was no longer convulsed; she spoke, but her talk was that of delirium. Had not the slightest appearance of syncope, for her face and lips were still very red, and her pulse was full and strong. She had leeches applied, and an evaporating lotion to the head, and active purgatives were given. The delirium continued two or three days, but she eventually recovered. The blood was taken in four cups, the first of which was buffed and cupped, but the other three not at all so.

Case IV. is the case of a child who had had scarlet fever succeeded by anasarca, and subsequently by pneumonia. I had been attending her about a week, without perceiving any symptom which would indicate a local affection, particularly of an acute character, when I was hastily called to see her, and found her propped up in bed, with quick, short, and painful breathing, a violent pain in the right side of the chest, and some tenderness in the region of the liver, but the pain was referred to a part higher up; there was a distressing dry cough; her pulse was very frequent and hard; her belly was slightly swollen, as well as her hands and feet; her urine was scanty and high-coloured, and her bowels were usually confined, unless acted upon by aperients. There had been slight difficulty of breathing the preceding day, with some pain in the side.

I thought this was a decided case of pneumonia, and that the liver was also inflamed. Her friends were told that it would be necessary to take away a *large* quantity of blood, as the only measure at all likely to afford her relief. As the blood flowed, her breathing became more and more relieved, till she could breathe with perfect ease. She lost seventeen ounces of blood without fainting. Her mother thought her almost well, she had derived so much benefit from the remedy.

I saw her at twelve o'clock the next day: she was breathing almost naturally, her pulse was not so frequent, she had no pain, and she was eating, with an apparent appetite, some baked potatoes and gravy, which her mother had imprudently given to her. In the evening of this day I was told (for I could not see her,) that her pain returned, her breathing became difficult and distressing, and these symptoms continued and increased till she died, at twelve o'clock the next day.

There were no symptoms subsequent to the bleeding that would lead me to suppose that she suffered in the least degree from the loss of blood.

The examination of the body, which took place twenty-four hours after death, was an interesting one, and I conducted it with great care and attention.

In the abdominal cavity was contained about four ounces of a transparent and slightly green fluid, in which were floating shreds of coagulable lymph. The intestines and stomach were of a natural colour, but the mucous membrane of the latter, in several places of the cardiac portion, was perforated, so that, upon holding it up to the light, it was perceived that its peritoneal coat alone kept in its contents, consisting of a small quantity of a darkish fluid, and some half-digested potato. There were some parts of the mucous membrane of the stomach redder than others; I believe not indicating disease having existed there, but it seemed such an appearance as the one described by Dr. YELLOWLY, and often found in a healthy state of that organ. It was much distended with air, and so were some portions of the bowels, particularly the transverse arch of the colon. The liver was of a natural structure and colour, but some part of its peritoneal covering was thickened and opaque. The other viscera in this cavity, as well as the pelvic viscera, were perfectly healthy. The abdominal vessels appeared to contain as much blood as I have usually found.

The thoracic viscera evinced extensive traces of disease. There was in the right cavity of the chest a small teacupful of fluid, similar to that in the abdomen, but more loaded with shreds and flakes of coagulable lymph. The left cavity contained rather more than half this quantity, but of the same character. Two thirds of the lower part of the right lung were hepatized; the pleura pulmonalis covering this part was beautifully injected with blood of a florid colour, and over it was spread a thin layer of lymph, of a light yellow colour, and attached in some places by shreds to the pleura costalis. These adhesions were evidently of recent date, as I could break them, so tender were they, by throwing a strong stream of water on them. Half the left lung appeared hepatized, but the pleura covering it was neither injected, as in the right lung, nor covered by lymph. Portions of the right lung sank in water, but it was not so with the left. There were hypertrophy and considerable enlargement of both the right and left ventricles of the heart; the auricles were also much dilated; the pericardium contained about half an ounce of fluid.

The brain was rather pale, but not more so than I have seen in some cases where no blood had been lost. There was a slight effusion between the pia mater and tunica arachnoides, and the latter membrane was in some parts rather opaque, so as to give the fluid seen through it somewhat of a milky appearance.

The subject of Case V. was a little boy, who had had measles; and it was on the fourth day from the commencement of the eruption that he was seized with tracheitis and pleuritis. I would merely observe here, that I think it is at this period that children with measles are more particularly liable to such inflammatory affections.

Case VI. was the case of a young woman suffering with violent pain in the front part of the head. When I saw her, she had been ill two days; her skin was exceedingly hot, her pulse 120, her tongue was œdematous and furred, and her breath fetid; there was delirium, with great aversion to light and noise: the latter was particularly distressing to her. I found that she had been subject to such attacks, but not always attended with pain in the head: sometimes the chest was the seat of pain, and at others the region of the liver. In all the former attacks, she told me that the alvine evacuations were "lumpy and dark coloured," and that the application of a few leeches, with opening medicine, either soon cured the pain, or caused it to move from one of those places already mentioned to the other. She was informed by her medical attendant that she had had inflammation of those various parts; and I recollect the time when I should have told her the same thing. Complete syncope was produced by the abstraction of seven ounces of blood, with little permanent relief to the pain; she, however, was much relieved when her bowels had been copiously acted upon.

Case VII. was precisely like the one I have just stated; and it will be observed that the loss of nearly the same quantity of blood produced fainting. Both these young women had exerted themselves more, and experienced more fatigue, the day before the attack, than what they had been accustomed to; and this circumstance may probably be considered as the immediate exciting cause of their illness.*

The case of hysteria, Case VIII., occurred in a young woman of a remarkable strong constitution, and she fainted at the loss of ten ounces of blood. I will just remark, that she was not bled during the hysterical paroxysm, but about two hours after one of these attacks.

I must here again insist upon the distinction between these inflammatory and non-inflammatory cases. The patient, Case VI. had had her attacks so repeatedly, that disorganization must have taken place, had they been

* I think no one can contrast these two cases with No. I. without being forcibly struck with the great difference as respects the susceptibility to the effect of loss of blood which these two different diseases had produced in each patient.

of the nature of inflammation. Dr. Gooch speaks of the irritable uterus; Sir Astley Cooper of the irritable breast; and Mr. Brodie of hysteria of the joints: all founding their opinions on this very circumstance. Dr. Hall has described similar affections of the head, chest, and abdomen, and has pointed out their distinctive characters of intolerance of loss of blood; and I have thus added my testimony to the truth of this author's very useful observations. These affections, however protracted, have no tendency to run into inflammation, so distinct are they from beginning to end. They do not bear the loss of blood. They are denoted by more urgent symptoms: the irritable brain by acute pain and intolerance of light; the irritable abdomen by acuter pain, but not so much tenderness.

P. S. I remark two observations in confirmation of these views, in two recent Numbers of the Lancet, by Dr. Alison and Dr. Elliottson. The former observes of a patient affected with pneumonia and pleuritis, "He had been bled to forty-six ounces, which, perhaps, in a young patient might be the average quantity necessary to subdue pneumonia;" and of the use of bloodletting in certain cases of painful dyspepsia,—"the quantity of blood generally necessary to be drawn is not great: he (Dr. A.) had known relief usually to follow the loss of twelve ounces; and Dr. Wilson Philip states the same."* Dr. Elliotson, speaking of a patient affected with peritonitis, says, "I had him bled to syncope, and thirty-two ounces of blood were abstracted before he fainted."† Indeed, such observations are incidentally made, continually, in medical writings.

---

